# What Matters to Patients With Nonsyndromic Craniosynostosis and Their Parents: A Qualitative Study Informing the Development of a Patient-Reported Outcome Measures Set

**DOI:** 10.1097/SCS.0000000000011491

**Published:** 2025-05-16

**Authors:** Pauline A.E. Tio, Coralie J. Wijnhoven, Elin Weissbach, Jacoba Kats, Jolanda M.E. Okkerse, Irene M.J. Mathijssen, Karolijn Dulfer

**Affiliations:** * Department of Plastic and Reconstructive Surgery and Hand Surgery, Erasmus University Medical Center; † Department of Child and Adolescent Psychiatry/Psychology, Erasmus University Medical Center, Sophia Children’s Hospital; ‡ Department of Neonatal and Pediatric Intensive Care, Division of Pediatric Intensive Care, Erasmus MC Sophia Children’s Hospital, Rotterdam, The Netherlands

**Keywords:** Craniosynostosis, patient-reported outcome measures, qualitative study (MESH)

## Abstract

The primary objective of this study was to identify important concepts that hold significance for both patients with nonsyndromic craniosynostosis and their parents regarding their craniosynostosis care. The findings from this study will guide the development of patient-reported outcome measures (PROM) sets tailored to specific craniosynostosis subtypes and age groups, which will be implemented in our treatment protocol. A qualitative methodology was used, and data were obtained through focus groups. A purposive sample of patients and parents of patients with nonsyndromic craniosynostosis was collected at Sophia Children’s Hospital Erasmus Medical Center. The focus groups were recorded, transcribed verbatim, and analyzed using thematic content analysis. Important domains in the preoperative and postoperative period were identified. In total, 34 participants were included in this study, of which 24 parents and 10 patients were divided over 12 focus groups. Based on our qualitative data, 4 top-level domains were considered to be of importance in patients with nonsyndromic craniosynostosis and their parents, including: emotional and social, cognitive, physical, and disease-specific functioning. Each top-level domain consisted of subdomains. Findings from this qualitative study reveal domains important to patients with nonsyndromic craniosynostosis and their parents with regard to their outcomes in craniosynostosis care. The comprehensive framework derived from this study serves as a guideline for developing a craniosynostosis-specific PROM set of relevance to our population.

Craniosynostosis is a rare congenital disorder characterized by the premature fusion of one or more cranial sutures, leading to distinct cranial deformities. In 2013, the prevalence of this condition in the Netherlands was reported at 7.2 per 10,000 live births.^1^ Approximately 70% of patients present with fusion of a single suture without any associated anomalies, which is known as nonsyndromic craniosynostosis.^2,3^ This group consists of sagittal, metopic, unicoronal, and unilambdoid synostosis. Individuals with nonsyndromic craniosynostosis constitute a heterogeneous population, being at risk for a spectrum of health problems such as elevated intracranial pressure, visual impairment, cognitive delays, and behavioral problems. Given these diverse challenges, assessing the impact of craniosynostosis on patients’ daily lives is crucial.

The impact on daily life is often assessed through patient-reported outcome measures (PROMs). PROMs are a key method for collecting patient-reported outcomes (PROs), which reflect a patient’s direct assessment of their health status, symptoms, and quality of life. They enhance the identification and communication of quality-of-life domains, providing valuable insights into patients’ experiences.^4–7^ Their integration into clinical practice supports a more patient-centered approach by helping health care providers identify specific concerns and align treatment decisions with individual health priorities. Consequently, PROMs facilitate better communication between patients and health care professionals, ensuring that care decisions reflect patients’ reported health status and health-related quality of life.^4,6,7^

Despite the well-documented benefits and widespread availability of PROMs for pediatric patients, their validation and use in craniosynostosis research remain notably limited. Existing literature reports heterogeneity in the use of PROMs to assess quality of life in patients with craniosynostosis.^8,9^ Currently, PROs are the least reported outcome in craniosynostosis studies,^8^ despite the proven efficacy of assessing PROs in pediatric care and the availability of pediatric PROMs.^6^ The suboptimal usage likely stems from 2 key factors. First, there is a limited number of PROMs that have been validated for use in patients with craniosynostosis. Second, no comprehensive research has been conducted to identify the most critical domains relevant for craniosynostosis patients and their parents. Measurement of the patient and parent perspective on domains that matter to them is essential to determine the impact of craniosynostosis on their daily lives. Without such data, the selection of appropriate PROMs remains inconsistent, further limiting their integration into clinical practice and research.

The underutilization and inconsistent application of PROMs in craniosynostosis research highlights the need for a more structured approach to their implementation. Given the acknowledged advantages of the use of PROMs in pediatric health care, a PROM set specific for patients with craniosynostosis is necessary. This study aims to employ qualitative methodology to identify those domains that are important for both patients with nonsyndromic craniosynostosis and their parents, with the objective of developing a comprehensive framework for a craniosynostosis-specific PROM set. The findings from this study will inform the development of PROM sets specific to craniosynostosis subtypes and age groups, which will be integrated into our clinical treatment protocol.

## METHODS

### Design

This study is a qualitative study based on focus group meetings with patients and parents of patients with nonsyndromic craniosynostosis. Focus groups facilitate discussion and introduce new perspectives, providing an opportunity to identify relevant outcomes and gain insight into the experiences and perceived needs of patients with craniosynostosis and their parents.^10^ In addition, focus groups have a positive effect on the interaction between participants and result in richer information.^11,12^ This study was approved by the local Ethics Committee (MEC-2022-0367). The Standards for Reporting Qualitative Research (SRQR) checklist was used for the reporting of this study.^13^

### Setting

This study was conducted at Sophia Children’s Hospital Erasmus Medical Centre, which is one of the 2 craniofacial centers in the Netherlands, with the focus group meetings taking place in an online format.

### Selection of Participants

Eligible participants for the online focus group meetings included patients with nonsyndromic craniosynostosis and their parents, all from Sophia Children’s Hospital Erasmus Medical Centre. Exclusion criteria were secondary craniosynostosis, syndromic craniosynostosis, and not mastering the Dutch language. A purposive sample was used. Recruitment occurred between August and December 2023. Eligible participants who visited our outpatient clinic received additional information about the study. Written informed consent was obtained from all participants. Patients and parents were divided into separate focus groups. The focus group meetings were divided into different age categories of the patients, as different domains and outcomes may be relevant in different age categories (0–12 and 12–18 y). A total of 12 focus group meetings were conducted with either patients with nonsyndromic craniosynostosis or their parents, see Supplemental Table 1, Supplemental Digital Content 1, http://links.lww.com/SCS/H867. Ideally, 3 to 5 participants were included in each focus group to foster a trusted environment and ensure that everyone had the opportunity to speak.

### Data Collection

Twelve focus group meetings were conducted between December 2023 and January 2024, facilitated by 2 researchers (P.A.E.T., female, MD and PhD candidate; and C.J.W., female, medical student). A topic guide was developed to structure the focus groups for both patients and their parents, drawing from relevant literature and clinical experience (Supplemental Digital Content 2, http://links.lww.com/SCS/H868). The focus groups were hosted on Microsoft Teams, with all participants being both visible and audible. Focus group meetings were recorded after permission of the participants. Separate discussions were held during the parent focus group meetings, focusing on both the preoperative and postoperative phases. Each focus group meeting lasted between 60 and 90 minutes. Field notes were taken during and after each session.

### Data Analysis

The recorded sessions were transcribed verbatim by one researcher (C.J.W.). Pseudonymisation was used to remove identifying characteristics. Transcripts were analyzed using Atlas.ti (Scientific Software Development GmbH, Berlin, Germany). A thematic content analysis on the data following an inductive approach was performed by 2 researchers (C.J.W. and P.A.E.T.) (Braun and Clarke, 2006).^14^ Transcripts were reviewed multiple times to assign codes and identify themes with both open coding and axial coding. In the last phase of selective coding, links between the categories were established, and all codes were categorized in top-level domains and subdomains. These (sub)domains were further refined through discussions within the multidisciplinary research team. The final outcome was a comprehensive framework of domains deemed important by patients with nonsyndromic craniosynostosis and their parents in relation to their outcomes from craniosynostosis care. Subsequently, the main findings were sent to all participants for participant validation.

## RESULTS

In total, 34 participants were included in this study, comprising 24 parents and 10 patients. Of the 24 parents who participated in this study, 20 were mothers (83%) and 4 were fathers (17%). Their children were diagnosed with scaphocephaly (n=6), trigonocephaly (n=8), anterior plagiocephaly (n=8), and posterior plagiocephaly (n=2). Of the 10 patients who participated in this study, 5 were female (50%) and 5 were male (50%). These patients were diagnosed with scaphocephaly (n=3), trigonocephaly (n=2), anterior plagiocephaly (n=3), or posterior plagiocephaly (n=2). The mean age of the patients at the time of the focus groups was 13.7 years. The participant characteristics are displayed in Supplemental Table 2, Supplemental Digital Content 1, http://links.lww.com/SCS/H867.

**FIGURE 1:**
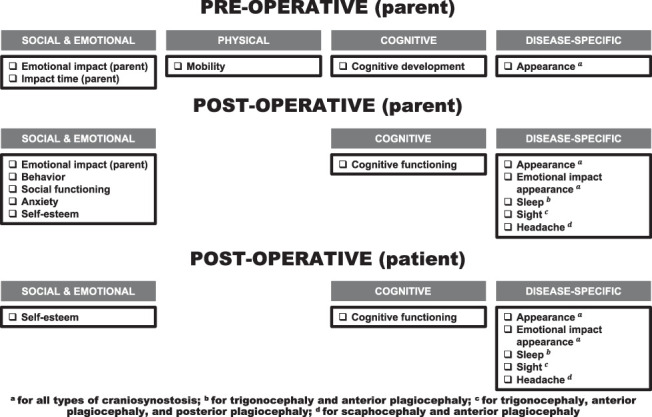
Framework of important domains to patients with nonsyndromic craniosynostosis and their parents.

Four top-level domains emerged from the focus groups, each encompassing subdomains, which together form the foundation of the comprehensive framework of important domains (Fig. 1). The analysis was divided into 2 periods: preoperative and postoperative. In the preoperative phase, 4 top-level domains emerged, including 5 subdomains. In the postoperative phase, 3 top-level domains emerged, including 11 subdomains. The primary domains and corresponding subdomains are discussed below, from the perspectives of both parents (preoperative and postoperative phase) and patients (postoperative phase).

### Social and Emotional

#### Emotional Impact on Parents

Both in the preoperative and postoperative phases, the diagnosis of their child's condition had a significant emotional impact on parents. Generally, most parents were the first to notice the abnormal head shape and sought medical advice. In some instances, they were referred to Sophia Children’s Hospital Erasmus Medical Centre, though often physicians reassured them that there was no cause for concern and recommended a wait-and-see approach. This left many parents feeling dismissed, with their concerns being downplayed or labeled as overprotective, leading to increased stress. The period between diagnosis and surgery was often described as particularly stressful and filled with anxiety. Many parents were uncertain about what to expect, and for some, receiving the diagnosis came as a profound shock. Upon reflection, the emotional toll during the preoperative phase emerged as the most significant subtheme, frequently emphasized in their accounts.

*“People tell you that you are being overly worried after giving birth, knowing that something isn’t right with your child. I knew it wasn’t right. You start researching: asymmetrical face. Then people tell you, “No, you’re worrying too much.” The doctors all say it’s nothing, and they suggest it might be hormones or something like that. But deep down, you know something really isn’t right. You feel quite alone. … once you hear the diagnosis, you just collapse.”* (*pa012ap-04, mother*)

*“It was like a rollercoaster. It was my first child, so I found it quite overwhelming. You’re referred to the craniofacial center and then things have to happen pretty quickly. … Looking back, I think it wasn’t that bad. But at the moment, you know, it’s such a small baby who has to undergo surgery, and I think it’s mainly hard for the parents.”* (*pa012sca-01*)*‬‬‬‬‬‬‬‬‬‬‬‬‬‬‬‬‬‬‬‬‬*‬‬‬

*“I thought it was really intense when I heard it [the diagnosis]. Yes, that really had an impact on us. ….. What I clearly remember is that your whole world falls apart. You thought it would never get better.”* (*pa012tri-01*)*‬‬‬‬‬‬‬‬‬‬‬‬‬‬‬‬‬‬‬‬‬*‬‬‬

*“Well, the road to it [the diagnosis] was also very grueling. There was a lot of stress. …. They called me an insecure mother who didn’t know how to handle her child. I was ridiculed by many. ”* (*pa012ap-01*)

The period immediately following surgery was also frequently described as highly stressful for parents. Although the emotional burden generally decreased in the years following the procedure, some parents continued to experience significant stress. This remaining emotional strain was often attributed to ongoing concerns about their child’s health and development, as well as fears regarding potential bullying from other children.

*“After the surgery, that was the most intense period for me, both before and just after it. …. I think we were more overwhelmed because the process moved very quickly for us—from diagnosis to surgery. In January, we got the diagnosis, and by mid-March, he was on the operating table. So, we were in a whirlwind, arranging everything and whatnot. But once it was all over, I thought, ‘Why did I worry so much?’ That feeling still lingers today. I’m super glad it’s all behind us.”* (*pa1218ap-02*)

*“I must say, the recovery went quite quickly. The day after the surgery, she was already trying to walk. She wanted to stand and walk everywhere. We were afraid she might bump her head, so we were very protective, but she didn’t seem bothered at all.”* (*pa1218pp-01*)

#### Impact on Time of the Parents

Although less intense than the emotional burden, the time commitment involved was also a notable concern in the preoperative phase. Parents reported dedicating significant time to seeking an accurate diagnosis, consulting various specialists, preparing for surgery, and attending follow-up appointments. While this had an impact on their daily lives, not all parents considered this aspect problematic, nor did it significantly impact other family members.

*“Well, yes, I think it definitely has a huge impact on your time, and also on work and everything. It obviously has an impact.”* (*pa012sca-01*)*‬‬‬‬‬‬‬‬‬‬‬‬‬‬‬‬‬‬‬‬‬*‬‬‬

#### Social Functioning

Several parents expressed concerns about their child’s social functioning in the years following surgery, noting that their children remained in the background in group settings and struggled with social interactions. In some cases, parents perceived their child as having difficulties forming new friendships, and some children received therapy to address these issues. An overarching pattern observed was that these problems became less pronounced as children grew older. However, none of the participating children themselves reported difficulties with social interactions, making new friends, or engaging with others.

*“We followed an introverted support program to help her build resilience because she’s very insecure. She has trouble standing up for herself, but that could just be her character.”* (*pa012sca-02*)

*“I think it’s important for a child to function well socially, and that’s a fear we have. … She really struggled to interact with other kids.”* (*pa012ap-02*)

#### Behavior

Behavioral problems were relatively common and often mentioned by parents, with several patients being diagnosed with ADHD or autism. These issues were particularly noted by parents of patients with trigonocephaly. The years leading up to a diagnosis were often described as frustrating and puzzling for parents. However, some parents attributed their child’s behavior to factors such as puberty or personality traits.

*“In the summer, our daughter was diagnosed with autism, after a period with struggles.”* (*pa1218tri-05*)

*“He also has ADHD, which brings its own challenges but also a lot of joy and energy. Unfortunately, during primary school, the support wasn’t great.”* (*pa1218tri-02*)

#### Anxiety

Parents frequently reported postoperative anxiety in their children, with anxiety peaking in the period immediately following surgery and gradually decreasing over time. Several parents attributed their child’s anxiety to posttraumatic stress disorder (PTSD), which in many cases improved following Eye Movement Desensitization and Reprocessing (EMDR) therapy. This therapy was provided at Sophia Children’s Hospital.

*“He had only slept 1.5 hours in 3 days and 3 nights and was having tantrums 24/7. Only then we contacted the hospital. This has become much better after the EMDR therapy.”* (*pa012ap-03*)

*“I think it is important to mention that he still has an extreme fear of dentists, doctors, orthodontists, and getting vaccinations. It’s complete panic, and that’s something worth noting.”* (*pa1218tri-01*)

#### Self-Esteem

Both parents and patients noted issues with low self-esteem. According to parents, this was often related to the child’s appearance; however, some patients also related this to their school performance.

*“I can see it at school and in his football team. He struggles with it [self-esteem]. I can’t pinpoint exactly what it is, but I think he could definitely have more self-esteem.”* (*pa012tri-01*)

*“Since our daughter turned 9, her self-esteem has really deteriorated.”* (*pa1218tri-03*)

*“[feeling insecure]… about different things, including my appearance and school.”* (*pt1218tri-02*)

### Physical

#### Mobility

In the preoperative phase, some parents expressed concerns regarding their child’s mobility. Some children required physiotherapy before surgery, particularly to support motoric development or address limited neck mobility, aiming to improve overall physical functioning.

*“My daughter was very immobile. For example, if she lay on her back, she just stayed there. She moved very little.”* (*pa1218tri-03*)*‬‬‬‬‬‬‬‬‬‬‬‬‬‬‬‬‬‬‬‬‬*‬‬‬

*“The only thing she still has is issues with her neck muscles because she had torticollis when she was born.”* (*pa1218pp-01*)*‬‬‬‬‬‬‬‬‬‬‬‬‬‬‬‬‬‬‬‬‬*‬‬‬

### Cognitive

#### Cognitive Development

Parents frequently expressed concerns about their child’s cognitive development, particularly in relation to the altered head shape and the potential risk of increased intracranial pressure. Even in cases where no developmental issues were evident at the time, parents continued to worry about their child’s future cognitive progress.

*“I was more focused on development because, for a long time, we didn’t know what was going on. …. No one could tell me what he actually had, so I was really focused on his development at that point.”* (*pa1218ap-02*)*‬‬‬‬‬‬‬‬‬‬‬‬‬‬‬‬‬‬‬‬‬*‬‬‬

#### Cognitive Functioning

Both parents and patients reported challenges with cognitive functioning in the child, particularly in the school setting. Difficulties were most often observed in areas such as learning, focusing, and attention. However, most patients felt that their struggles were not significantly more pronounced than those of their peers.

*“With my son there is a discrepancy. He is cognitively very strong, but has a very slow processing time, which in turn causes problems.”* (*pa012ap-04*)

*“I can definitely relate to the concentration issues. … He struggled a lot with concentration and had homework supervision for a while, but he doesn’t need that anymore.”* (*pa1218sca-03*)

*“When I’m in class, I can generally keep my focus, but sometimes I get distracted by others.”* (*pt1218ap-01*)

### Disease-Specific

#### Appearance

Appearance emerged as a major theme during both the preoperative and postoperative phases across all types of craniosynostosis, reported by both parents and patients. All parents noticed some form of abnormality in their child’s appearance after birth, although the severity varied. In all cases, concerns about the appearance were the primary reason for seeking a referral.

*“You could clearly see that her head was growing towards the right. That side was much rounder. The back was completely flat. One ear and one eye weren’t aligned. You could definitely see it.”* (*pa1218pp-01*)*‬‬‬‬‬‬‬‬‬‬‬‬‬‬‬‬‬‬‬‬‬*‬‬‬

*“His forehead kept protruding, and he had a very narrow skull on the sides. It was really like a boat-shaped skull. It was very noticeable.”* (*pa1218sca-02*)*‬‬‬‬‬‬‬‬‬‬‬‬‬‬‬‬‬‬‬‬‬*‬‬‬

*“It [the craniosynostosis] was very visible in my child. She had a much narrower forehead, and there was a significant ridge on her forehead. It was really obvious.”* (*pa1218tri-03*)*‬‬‬‬‬‬‬‬‬‬‬‬‬‬‬‬‬‬‬‬‬*‬‬‬

Postoperatively, parents often highlighted issues such as abnormal head shape or visible scars. While most scars typically blend into the hairline, some parents reported that they remained noticeable and required additional attention. Concealing these scars became a common coping strategy. Although not all patients felt negatively about their appearance, it remained a recurring concern and continued to be a prominent issue for many.

*“You can still really see it… He has a very large forehead, his eyebrows are completely sunken in, and his head is still quite crooked, especially in photos. His ears and nose are also misaligned. Even after another surgery, it’s still quite noticeable. When people take pictures, they ask if he could straighten his head, but of course, he can’t.”* (*pa012ap-04*)

*“I have a scar from ear to ear, but you can hardly see it. I don’t mind others noticing it now, though I was more self-conscious about it when I was younger.”* (*pt1218sca-03*)

*“The gel should be placed on the hair in the opposite direction because then you cannot see the scar very well.”* (*pa1218sca-01*)

*“His head isn’t perfect. His ears are still a bit crooked, and his eyes are too. You mainly see it in the mirror. You feel bumps on the back of his head.”* (*pa012pp-01*)

#### Emotional Impact of Appearance

Both parents and patients acknowledged the emotional toll related to appearance. Some parents expressed concerns about the emotional impact on their child, particularly in cases where the child had experienced bullying due to their appearance. While only a few patients reported feeling truly insecure, many were preoccupied with the appearance of their head or scars, leading them to adopt specific hairstyles to conceal them.

*“Our daughter was bullied a lot in primary school because of her narrow head. It runs in the family, but her scar made it worse. The scar line was placed a bit too far back, so whenever her hair gets even slightly greasy, the scar becomes visible. She’s not bullied for it anymore, but she really struggled with it at the time.”* (*pa1218tr-05*)

*“I sometimes feel insecure about my appearance. A few months ago, it was worse, but now it’s getting better. I still find it difficult sometimes.”* (*pt1218sca-03*)

*“When he was younger, it [the scar] was an issue. Being a boy, he often had short hair, but he never wanted it too short because of that thick scar. He didn’t want everyone to see it. That was an issue.”* (*pa1218ap-02*)

#### Sleep

Concerns about sleep were particularly noted in patients with anterior plagiocephaly and trigonocephaly, manifesting as difficulties falling asleep and, in some cases, sleep apnea. Some younger patients underwent a polysomnography to objectively assess sleep disturbances and determine the need for treatment. Despite these challenges, neither patients nor parents reported daytime issues related to lack of sleep.

*“He’s had sleep apnea, so his tonsils and adenoids were removed. We noticed it when he was in the car, sleeping in the back seat. You wouldn’t hear anything for a while, then suddenly, a loud snore—he would be back again”* (*pa012tri-02*)

*“Yes, it’s very difficult for me to fall asleep, really, but I also take melatonin, otherwise I really can’t fall asleep.”* (*pt1218ap-03*)

#### Sight

Vision problems were frequently reported by both parents and patients, particularly in cases of trigonocephaly, anterior, and posterior plagiocephaly. Many patients required glasses and were receiving ongoing treatment from an ophthalmologist. Even in cases where no vision problems were present, parents expressed significant concerns about the potential for future eye issues.

*“He had a lazy eye, which later needed corrective support. He got glasses at a later age.”* (*pa1218ap-02*)

*“She had an eye condition, and from day one, we wondered if it could be connected. She even saw an ophthalmologist for a while, struggling with what I believe was focusing or accommodating, though I’m no expert. She wore glasses temporarily, and it seems fine now.”* (*pa1218tri-04*)

#### Headache

Both patients and parents identified headaches as a concern in patients with scaphocephaly and anterior plagiocephaly. Parents were primarily concerned because of the possible relation to increased intracranial pressure. However, headaches typically emerged at a later age, with patients attributing them to other causes.

*“I’m noticing that now she gets older, she’s getting headaches more frequently. It’s not that the headaches are worse, but they happen more often. I can’t help but wonder if it’s connected [to the craniosynostosis]. Everyone gets headaches, but could it be related? Or maybe the scar is pulling, and she describes it as a headache.”* (*pa012sca-02*)

## DISCUSSION

The integration of PROMs in routine pediatric care enables health care providers to detect issues across various health-related quality of life domains, thereby improving communication and outcomes.^4^ To improve health-related quality of life, it is crucial to measure patient and parent perspectives on the concepts that matter most to them. Patients with nonsyndromic craniosynostosis can face a variety of challenges, and craniosynostosis care involves comprehensive follow-up and diverse surgical techniques. In other disease populations, discussing PROMs in the outpatient clinic has proven effective in delivering care that aligns with patient’s or family’s needs, focusing on the most significant concerns, improve clinical outcomes.^4,15,16^ To our knowledge, this is the first study to define key domains important to patients with nonsyndromic craniosynostosis and their parents in relation to outcomes in craniosynostosis care. This study provides a detailed framework to guide the development of a craniosynostosis-specific PROM set from both the patient and parent perspectives. Based on qualitative data gathered in several focus groups comprises 4 top-level domains covering outcomes important to both patients with non-syndromic craniosynostosis and their parents: social and emotional, cognitive, physical, and disease-specific functioning.

The emotional impact on parents was a significant theme in the focus groups with parents. Most parents reported substantial emotional distress during both the preoperative and postoperative phases. In the preoperative phase, stress and anxiety were prevalent, primarily driven by uncertainty about the diagnosis and the prospect of surgery. This aligns with prior research, which emphasizes the emotional burden on parents during this time.^17,18^ In addition, many parents felt that the diagnostic process was prolonged, further exacerbating their anxiety. Consistent with our findings, most parents felt a sense of relief postoperatively, believing the most challenging phase had passed, though new concerns often emerged. In the immediate postoperative phase, these concerns typically revolved around the child’s recovery. However, the emotional intensity usually peaked shortly after surgery and gradually diminished over time. As their child matured, parents’ focus shifted toward concerns about development and specific needs, a trend also noted in earlier studies.^17,19^ Both parents and young adolescent patients expressed a strong desire for peer support groups, both before and following surgery, but often struggled to find appropriate support networks, especially since most members of the national patient association have a syndromic type of craniosynostosis. Peer support is widely acknowledged as highly valuable,^20^ with parents of children with craniosynostosis often considering it the most helpful form of support.^18,19^ Such peer interactions play a crucial role in helping parents cope with and understand their child’s diagnosis.^20^

The impact of the child’s appearance on both parents and patients was another prominent topic. Many patients reported being preoccupied with their appearance, particularly with regard to their face and scar. Patients tend to be more affected by their appearance than their parents.^21^ Therefore, incorporating PROMs focused on appearance, particularly in adolescents, is important for improving treatment protocols and enhancing quality of life.

Several key domains identified in this study align with research on nonsyndromic craniosynostosis. Our findings provide additional detail and complement existing literature. Notably, our findings highlight the importance of monitoring anxiety in children, as many parents reported postoperative anxiety in their children, often attributed as PTSD. This anxiety was frequently alleviated through EMDR therapy, which was offered at Sophia’s Children’s Hospital. Some children were diagnosed with PSTD later, resulting in severe issues, such as tantrums, sleep disturbances, and difficulties at places like the dentist or hairdresser. Literature on various pediatric populations shows an increased risk of developing PTSD after surgery.^22–24^ Turgoose et al^24^ found a prevalence of 16% for PTSD in pediatric surgical patients, higher than in the general population. Therefore, monitoring anxiety in patients with craniosynostosis is essential for quality of life for both patients and families.

Self-esteem emerged as a recurring theme in adolescence for both parents and patients with nonsyndromic craniosynostosis, despite literature reporting comparable self-esteem scores between these patients and the normative population.^25,26^ This discrepancy suggests that while patients may not actually have a lower self-esteem, the concept itself holds importance for them and their families. The increased parental stress and concerns associated with craniosynostosis may lead to a preoccupation with the child’s abilities and worth.^18^ This parental focus could, as a result, influence the child’s own perception and emphasis on self-esteem.^27^ The interplay between parental attitudes and adolescent self-concept in the context of craniosynostosis warrants further investigation to improve understanding of this complex dynamic.

A strength of this study is the inclusion of participants from different types of nonsyndromic craniosynostosis and varying age groups. The diversity allowed us to capture a broad range of perspectives. All focus groups were conducted by the same researchers, who were not involved in the care of the participants, which helped ensure objectivity. Qualitative research provides valuable insights into the experiences of parents and patients, allowing them to share their perspectives in a way that surveys cannot replicate. However, this study has limitations. First, some parents’ experiences were based on surgeries that took place up to 17 years ago, which may have led to recall bias. To reduce this effect, we chose focus groups, as they allow participants to refresh each other’s memories. Second, due to the rarity of posterior plagiocephaly, only 2 parents and 2 patients participated, and they were interviewed separately rather than in focus groups.

Future research will focus on identifying important domains for patients with syndromic craniosynostosis and their parents in relation to craniosynostosis care outcomes. This will lead to the development of a comprehensive framework for each syndromic craniosynostosis group, facilitating craniosynostosis-specific PROM sets. In addition, research will explore the impact of implementing this PROM set in clinical practice for patients with craniosynostosis.

In conclusion, findings from this qualitative study reveal important domains for patients with nonsyndromic craniosynostosis and their parents regarding craniosynostosis care outcomes. The comprehensive framework derived from this study will guide the development of a craniosynostosis-specific PROM set relevant to our patient population.

## Supplementary Material
