# Supplementary material for: What Matters to Patients With Nonsyndromic Craniosynostosis and Their Parents: A Qualitative Study Informing the Development of a Patient-Reported Outcome Measures Set
Source: J Craniofac Surg. 2025 May 16;36(7):2320–6. doi: 10.1097/SCS.0000000000011491 (PMC12502935; doi:10.1097/SCS.0000000000011491)
Supplement: Supplementary file 1 [file scs-36-02320-s001.docx]

**SUPPLEMENTAL Table 1.** Division of focus groups

| **Focus group** | **Age patient (years)** | **Participants** | **Type of craniosynostosis** |
| --- | --- | --- | --- |
| 1 | 0-12 | Parents | Scaphocephaly |
| 2 | 0-12 | Parents | Trigonocephaly |
| 3 | 0-12 | Parents | Anterior plagiocephaly |
| 4 | 0-12 | Parents | Posterior plagiocephaly |
| 5 | 12-18 | Parents | Scaphocephaly |
| 6 | 12-18 | Parents | Trigonocephaly |
| 7 | 12-18 | Parents | Anterior plagiocephaly |
| 8 | 12-18 | Parents | Posterior plagiocephaly |
| 9 | 12-18 | Patients | Scaphocephaly |
| 10 | 12-18 | Patients | Trigonocephaly |
| 11 | 12-18 | Patients | Anterior plagiocephaly |
| 12 | 12-18 | Patients | Posterior plagiocephaly |

**SUPPLEMENTAL Table 2.** Participant characteristics

|  | n (%) |
| --- | --- |
| **Total Participants** | 34 |
| Parents | 24 |
| Patients | 10 |
| **Total Parents** | 24 |
| Mother | 20 (83%) |
| Father | 4 (17%) |
| **Diagnosis of their child** |  |
| Scaphocephaly | 6 |
| Trigonocephaly | 8 |
| Anterior plagiocephaly | 8 |
| Posterior plagiocephaly | 2 |
| **Total Patients** | 10 |
| Female | 5 (50%) |
| Male | 5 (50%) |
| **Age Patients (in years)** |  |
| Mean | 13.7 [range 12-18] |
| **Diagnosis Patients** |  |
| Scaphocephaly | 3 |
| Trigonocephaly | 2 |
| Anterior plagiocephaly | 3 |
| Posterior plagiocephaly | 2 |
