# Supplementary material for: What Matters to Patients With Nonsyndromic Craniosynostosis and Their Parents: A Qualitative Study Informing the Development of a Patient-Reported Outcome Measures Set
Source: J Craniofac Surg. 2025 May 16;36(7):2320–6. doi: 10.1097/SCS.0000000000011491 (PMC12502935; doi:10.1097/SCS.0000000000011491)
Supplement: Supplementary file 2 [file scs-36-02320-s002.docx]

**Supplement 1: Topic Guides ^a^**

^a^ Topic guides were originally in Dutch but translated to English for publication

**Topic Guide Parent Focus Group**

Welcome to this focus group about the experiences in the care of patients with craniosynostosis. The researchers will introduce themselves with their name and function. First, we are very happy that you want to participate in this focus group and for that we want to thank you.

[...]

I would like to explain briefly what the purpose of the research is and why we are doing this. We would like to hear what matters to you as a parent with a child with craniosynostosis. We would like you to tell stories to help us understand how it’s been as a parent with a child with craniosynostosis. Particularly, we are interested in how you feel about your child’s care, the healthcare for patients with craniosynostosis, and other related issues. We are interested in both the pre-operative period and the post-operative period. We would like to know how the diagnosis of your child affected your child and your family and how we can improve treatment. By discussing this with you in a focus group, we hope to gain more clarity about what is going on with you and what is important to you.

This meeting will last approximately one hour to one and a half. An audio-recording is made of this focus group, as indicated in the information pack. This recording is made so we are able to transcribe and process the discussion properly. These transcripts will not contain your names and will not be traceable to individuals.

Before we proceed, are there any questions?

[…]

First, can you tell us something about yourself and your child?

How were your experiences with a child with craniosynostosis?

How were your experiences with the care provided at Sophia Children’s Hospital?

Can you tell us about the surgery?

[…]

Now we will focus on the pre-operative period. What was important to you?

Can you tell me about the cognitive functioning and development of your child?

Can you tell me about the social impact and aspects of having a child with craniosynostosis (parent/family/child)?

Can you tell me how your child is functioning social?

Can you tell me how your child is functioning emotional?

Can you tell me how your child is functioning physical?

Can you tell me about disease-specific problems of your child?

How often did these problems occur in the pre-operative period? And how often did you visit the hospital with your child?

[…]

Now we will focus on the post-operative period. What was important to you?

Can you tell me about the cognitive functioning and development of your child?

Can you tell me about the social impact and aspects of having a child with craniosynostosis (parent/family/child)?

Can you tell me how your child is functioning social?

Can you tell me how your child is functioning emotional?

Can you tell me how your child is functioning physical?

Can you tell me about disease-specific problems of your child?

How often are there problems? And how often do you visit the hospital with your child?

Are you satisfied with the treatment you’ve had for your child’s craniosynostosis?

How would you change the treatment you have had?

Before we end this focus group, is there a certain subject or specific topic during this focus group that is of importance but has not yet been discussed? […] Are there any last questions?

[…]

We would like to thank you very much for all your input during this focus group.

**Topic Guide Patient Focus Group**

Welcome to this focus group about the experiences in the care of patients with craniosynostosis. The researchers will introduce themselves with their name and function. First, we are very happy that you want to participate in this focus group and for that we want to thank you.

[...]

I would like to explain briefly what the purpose of the research is and why we are doing this. We would like to hear what matters to you as a patient with craniosynostosis. We would like you to tell stories to help us understand how it’s been as a child with craniosynostosis. Particularly, we are interested in how you feel about your care, the healthcare for patients with craniosynostosis, and other related issues. We are interested in how the diagnosis affected you and how we can improve treatment. By discussing this with you in a focus group, we hope to gain more clarity about what is going on with you and what is important to you.

This meeting will last approximately one hour. An audio-recording is made of this focus group, as indicated in the information pack. This recording is made so we are able to transcribe and process the discussion properly. These transcripts will not contain your names and will not be traceable to individuals.

Before we proceed, are there any questions?

[…]

First, can you tell us something about yourself? How old are you? What kind of activities do you like?

Can you tell me about the details of you craniosynostosis?

What treatments have you had?

Do you like going to school? How is it going at school?

How is your concentration?

Can you tell us about your friends?

Can you tell us about your social life?

Is it easy to meet new people or make friends?

Can you tell us about your relationship with your family members?

Can you tell us about your physical functioning?

Do you have or have you had any problems for which you need to see a doctor? How does this affect you?

How often do you see a team member of the craniosynostosis care team?

How do you feel about your appearance?

How do you think others feel about your appearance?

Are you satisfied with the treatment you’ve had for your craniosynostosis?

How would you change the treatment you have had?

Before we end this focus group, is there a certain subject or specific topic during this focus group that is of importance but has not yet been discussed? […] Are there any last questions?

[…]

We would like to thank you very much for all your input during this focus group.
